# Clinical strategy of repeat biopsy in patients with atypical small acinar proliferation (ASAP)

**DOI:** 10.1038/s41598-021-02172-8

**Published:** 2021-11-30

**Authors:** Hwanik Kim, Jung Kwon Kim, Gheeyoung Choe, Sung Kyu Hong

**Affiliations:** 1 Department of Urology, Seoul National University Bundang Hospital, Seongnam, Korea; 2 Present Address: Department of Urology, Seoul National University College of Medicine, Seoul, Korea; 3 Department of Pathology, Seoul National University Bundang Hospital, Seongnam, Korea

**Keywords:** Cancer screening, Urological cancer, Prostate cancer, Cancer, Urology, Prostate

## Abstract

Atypical small acinar proliferation (ASAP) occurs in approximately 5% of prostate biopsies. Approximately 30–40% of patients with ASAP have biopsy detectable prostate cancer (PCa) within 5 years. Current guidelines recommend a repeat biopsy within 3–6 months after the initial diagnosis. The aim of the present study was to examine the association between ASAP and subsequent diagnosis of clinically significant PCa (csPCa). The need for immediate repeat biopsy was also evaluated. We identified 212 patients with an ASAP diagnosis on their first biopsy at our institution between February 2006 and March 2018. Of these patients, 102 (48.1%) had at least one follow-up biopsy. Clinicopathologic features including rates of subsequent PCa and csPCa were assessed. Thirty-five patients subsequently underwent radical prostatectomy (RP). Their pathologic results were reviewed. csPCa was defined as the presence of Gleason score (GS) ≥ 3 + 4 in ≥ 1 biopsy core. Adverse pathology (AP) was defined as high-grade (primary Gleason pattern ≥ 4) or non-organ-confined disease (pT3/N1) after RP. Of 102 patients, 87 (85.3%), 13 (12.7%), and 2 (2.0%) had one, two, and three follow-up biopsies, respectively. Median time from the initial ASAP diagnosis to the 2nd follow-up biopsy and the last follow-up biopsy were 21.9 months (range 1–129 months) and 27.7 months (range 1–129 months), respectively. Of these patients, 46 (45.1%) were subsequently diagnosed with PCa, including 20 (19.6%) with csPCa. Only 2 (2.0%) patients had GS ≥ 8 disease. Five (4.9%) patients had number of positive cores > 3. Of 35 patients who subsequently underwent RP, seven (20%) had AP after RP and 17 (48.6%) showed GS upgrading. Of these 17 patients, the vast majority (16/17, 94.1%) had GS upgrading from 3 + 3 to 3 + 4. 45.1% of patients with an initial diagnosis of ASAP who had repeat prostate biopsy were subsequently diagnosed with PCa and 19.6% were found to have csPCa. Our findings add further evidence that after a diagnosis of ASAP, a repeat biopsy is warranted and that the repeat biopsy should not be postponed.

## Introduction

Over the last 10 years, prostate cancer (PCa) incidence has gradually increased in Asian countries^1^. In general, a diagnostic prostate biopsy yields confirmatory results regarding the presence of PCa. In a minority of cases, however, the conclusion is less definitive, leading to a diagnosis like or atypical small acinar proliferation (ASAP). While an initial prostate biopsy is often performed in response to elevated prostate specific antigen (PSA) or abnormal digital rectal exam (DRE), factors that drive repeat biopsy include rising PSA and pathological findings associated with an increased risk of PCa^2^.

ASAP was first defined by Montironi et al.^3^ as “a focus of small acinar structures formed by atypical epithelial cells” having some but not all features of cancer^4^. Therefore, it is a histological diagnosis (not a disease) of exclusion for cases where suspicious features are present but inadequate to fulfil the diagnostic criteria for PCa^5^. It occurs in approximately 5% of prostate biopsies. Approximately 30–40% of patients with ASAP have biopsy detectable prostate cancer within 5 years^6,7^. Current guidelines recommend that an extended pattern repeat biopsy with focus at the initially positive site should be performed within 3–6 months of the initial ASAP diagnosis^8,9^. The aim of this study was to evaluate the association between ASAP and subsequent diagnosis of clinically significant PCa (csPCa). The need for immediate repeat biopsy was also evaluated.

## Materials and methods

After obtaining approval from Seoul National University Bundang Hospital Institutional Review Board approval (SNUBH IRB No: B-2010-643-102), our single-institutional prostate cancer registry and our clinically maintained prostate biopsy database containing data of patients between February 2006 and March 2018 were reviewed. MRI-guided fusion prostate biopsy started in 2015. All study protocols were in accordance with the principles of the Helsinki Declaration. We removed personal identifiers and anonymized all data, which exempted the study from the need to obtain informed consent from patients also approved by SNUBH IRB. Indications of a primary prostate biopsy included elevated PSA (≥ 3 ng/mL) or abnormal findings in DRE or ultrasound. Indications for a repeat biopsy are as follows: (1) Rising and/or persistently elevated PSA levels (the most common reason), (2) Suspicious DRE not previously detected in the timing of 1st biopsy, (3) New suspicious lesion in subsequent prostate MRI. Clinical data including patient demographics, age at biopsy, body mass index (BMI), date of biopsy, latest PSA at the time of biopsy, digital rectal examination findings, prostate volume by ultrasound, pathologic results of biopsy (e.g., sum of Gleason score and ASAP, or benign diagnoses), the time from the last prostate biopsy to subsequent repeat biopsy, detection rates of PCa, csPCa, and PCa with AP after robot-assisted laparoscopic radical prostatectomy (RALP), and Gleason score (GS) upgrading were recorded prospectively. Patients who were diagnosed as ASAP without PCa from ultrasound-guided prostate biopsy with a follow-up period of more than one year were included. Patients were excluded if they had previously received a diagnosis of prostate cancer or any kind of hormonal treatment except for 5a-reductase inhibitor. CsPCa was defined as the presence of Gleason score (GS) ≥ 3 + 4 in ≥ 1 biopsy core. Adverse pathology (AP) was defined as high-grade (primary Gleason pattern ≥ 4) or non-organ-confined disease (pT3/N1) after RP. Clinically insignificant prostate cancer was defined as GS 6.

Primary outcomes were PCa and csPCa detection rates at subsequent biopsy. Secondary outcomes were detection rates of PCa with AP and Gleason score upgrading in radical prostatectomy specimen pathology. Clinicopathologic features were evaluated through comparative analysis. 9980 patients underwent primary prostate biopsy and 212 (2.1%) patients were diagnosed as ASAP. Of 212 patients with ASAP, 102 (48.1%) had at least one follow-up biopsy. 110 patients could not receive repeat biopsy due to patients’ refusal, or high risk of biopsy-related complications, or follow-up loss. Among these 102 patients, 35 subsequently underwent radical prostatectomy (RP). Their pathologic results were reviewed. All biopsy specimens, either from biopsy or operation, were reviewed by fellowship trained genitourinary pathologists supervised by a single pathologist (G.C.) with experience in urologic pathology for more than 15 years. If the diagnostic discrepancy occurred between fellowship-trained genitourinary pathologists and G.C., they gathered together in one place to review the slides together and come to a consensus. The diagnosis of ASAP was confirmed by G.C. throughout the study period. α-Methyl acyl-CoA racemase (AMACR), high molecular weight cytokeratin (HMWCK), p63, and cytokeratin 5/6 (CK 5/6) were used as immunohistochemical antibody cocktail staining to assess the presence of basal cells and diagnose ASAP (02/2006–12/2015: AMACR + HMWCK + p63, 2016/01–2018/03 : AMACR + HMWCK + p63 + CK 5/6)^10^.

Clinical and treatment parameters of these 102 patients were analyzed statistically. Comparative analyses between groups after repeat biopsy (stratified into cohorts depending on whether any PCa was detected and whether any csPCa was diagnosed) were performed. Moreover, comparative analyses between groups after radical prostatectomy (stratified into cohorts depending on whether PCa with AP was diagnosed and whether GS upgrading was found) were conducted. In addition to descriptive statistics, we used the *χ*^2^ test for comparing categorical variables and the independent *t* test or Wilcoxon rank-sum test for comparison of continuous variables. Statistical significance was set at *p* ≤ 0.05 using SPSS version 22.0.

## Results

**Figure 1:**
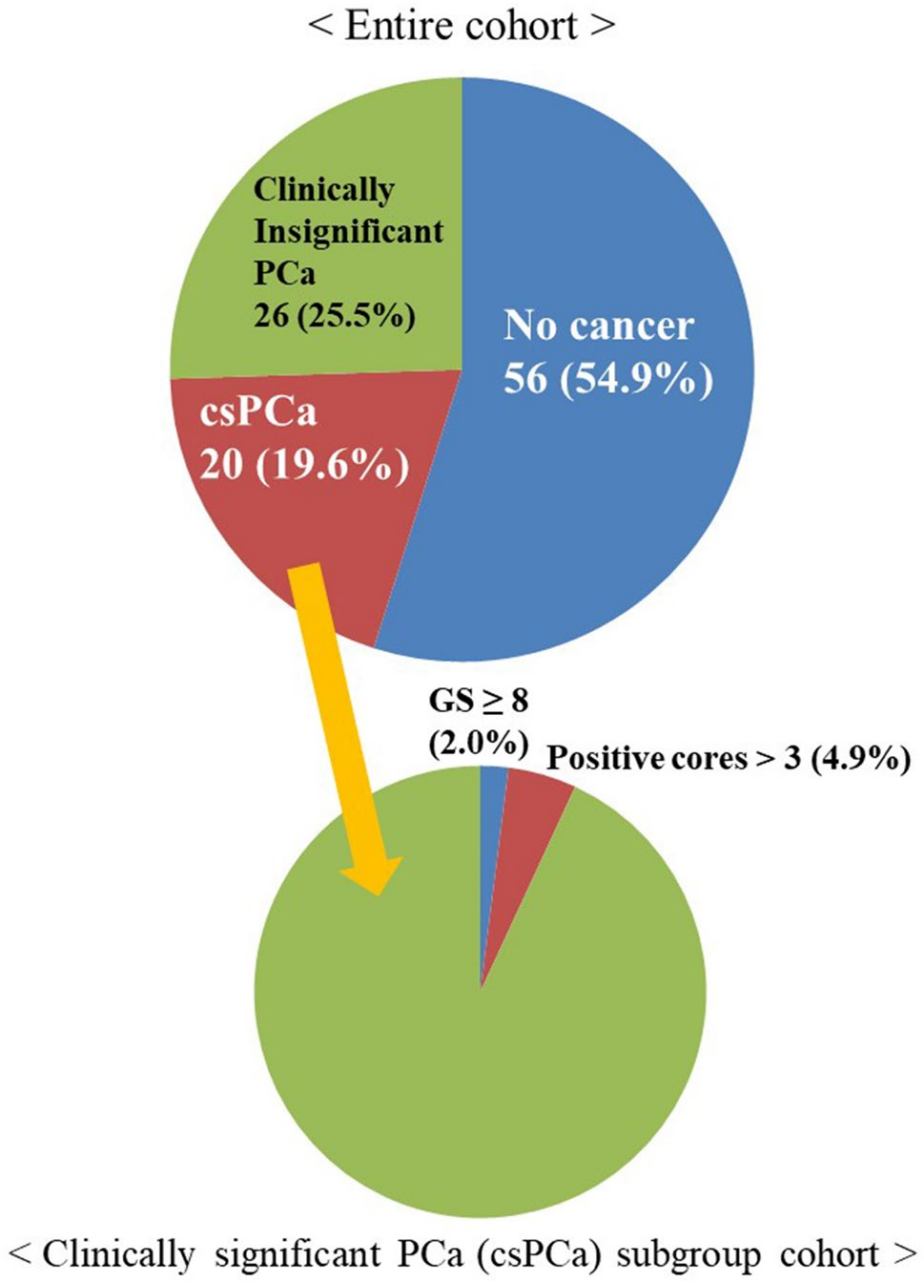
Patients with subsequent biopsy results.

Baseline characteristics between repeat biopsy group and non-repeat biopsy group are detailed in Supplementary Table S1. Repeat biopsy group had significantly more MRI Fusion biopsy (18.5% vs. 4.5%, *p* = 0.001) and more suspicious nodules at prostate MRI (17.6% vs. 3.6%, *p* < 0.001). Of 102 patients with repeat biopsy, the median time from the initial ASAP diagnosis to the 2nd follow-up biopsy was 21.9 months (range 1–129 months) and median time to the last follow-up biopsy was 27.7 months (range 1–129 months). Eighty-seven, 13, and 2 had undergone 2, 3, and 4 biopsies, respectively. Subsequent biopsy results of 102 patients were as follows. Forty-six (45.1%) patients were subsequently diagnosed with PCa, including 20 (19.6%) with csPCa (Fig. 1). Nine were re-diagnosed as ASAP. The rest had neither PCa nor ASAP (no abnormal pathologic finding). PCa was diagnosed for 40 patients on the 2nd biopsy, 5 on the 3rd biopsy, and 1 on the 4th biopsy. The distribution of GS for 46 patients was as follows: 26 for GS 3 + 3, 12 for GS 3 + 4, 6 for GS 4 + 3, 1 for GS 4 + 5, and 1 for GS 5 + 4. Among the entire cohort, only 2 (2.0%) patients had GS ≥ 8 and 5 (4.9%) had more than three positive cores.

Patients who were diagnosed with any PCa on repeat biopsy were older with higher BMI, higher initial prebiopsy PSA, lower PSA density (PSAD), and shorter mean time to the last repeat biopsy than those with negative biopsy results without showing statistical significance (Table 1). Patients who were diagnosed with csPCa on repeat biopsy were significantly older (*p* = 0.035) with marginally but significantly higher BMI compared to those without csPCa (*p* = 0.054). Other factors did not differ significantly between groups (*p* > 0.05; Table 1).

**Table 1:** Comparative analysis between groups after repeat biopsy.

Mean ± SD	Any cancer on repeat biopsy		*p* value	Clinically significant cancer on repeat Bx		*p* value
	Yes (N = 46)	No (N = 56)		Yes (N = 20)	No (N = 82)	
Age	65.9 ± 6.3	64.1 ± 8.0	0.206	68.0 ± 6.7	64.1 ± 7.3	0.035
BMI	24.6 ± 2.5	24.0 ± 2.6	0.287	25.4 ± 2.7	24.0 ± 2.5	0.054
Initial PSA	7.96 ± 5.80	7.80 ± 5.06	0.879	9.45 ± 6.56	7.47 ± 5.01	0.143
PSAD	0.19 ± 0.11	0.20 ± 0.16	0.576	0.20 ± 0.10	0.19 ± 0.15	0.744
Time to the last repeat Bx (months)	26.7 ± 27.2	28.5 ± 27.7	0.745	31.5 ± 27.8	26.8 ± 27.3	0.496

*SD* standard deviation, *BMI* body mass index, *PSA* prostate specific antigen (ng/mL), *PSAD* prostate specific antigen density (ng/mL/cc), *Bx* prostate biopsy.

Among 46 PCa patients, 35 underwent RALP. Of these 35 patients, 7 (20%) had AP and 17 (48.6%) showed GS upgrading in RALP specimen. Detailed information on 7 patients with AP is as follows: one patient had GS 4 + 3 on pT3aN0, one patient had GS 4 + 5 on pT3aN0, one patient had GS 3 + 4 on T3aN0, and four patients had GS 4 + 3 on pT2N0. Patients with AP in RALP specimen had significantly longer mean time to the last repeat biopsy (*p* = 0.023) than patients without AP. The vast majority (16/17, 94.1%) of patients with GS upgrading had upgraded from 3 + 3 to 3 + 4 (Table 2).

**Table 2:** Comparative analysis between groups after radical prostatectomy.

Mean ± SD	AP cancer after RALP		*p* value	GS upgrading after RALP		*p* value
	Yes (N = 7)	No (N = 28)		Yes (N = 17)	No (N = 18)	
Age	66.6 ± 2.6	65.6 ± 6.5	0.564	65.1 ± 6.4	66.5 ± 5.6	0.499
BMI	25.0 ± 3.7	24.2 ± 2.6	0.550	23.4 ± 2.4	25.1 ± 2.9	0.108
Initial PSA	6.43 ± 3.02	7.67 ± 6.01	0.603	5.66 ± 2.45	8.98 ± 6.95	0.071
PSAD	0.14 ± 0.08	0.18 ± 0.12	0.360	0.15 ± 0.11	0.20 ± 0.12	0.214
Time to the last repeat Bx (months)	63.3 ± 38.1	20.1 ± 20.2	0.023	30.1 ± 30.5	27.4 ± 29.3	0.795

*AP* adverse pathology, *RP* radical prostatectomy, *SD* standard deviation, *RALP* robot-assisted laparoscopic radical prostatectomy, *BMI* body mass index, *PSA* prostate specific antigen (ng/mL), *PSAD* prostate specific antigen density (ng/mL/cc), *Bx* prostate biopsy.

## Discussion

The clinical management of ASAP still represents a dilemma for urologists. ASAP indicates the presence of suspicious glands with insufficient cytological or architectural atypia for a definitive diagnosis of prostatic adenocarcinoma. In short, it indicates a situation of diagnostic uncertainty, 30–40% to be PCa on subsequent biopsies^11^. Although ASAP was diagnosed only about 2% of approximately 10,000 patients with prostate biopsies over a decade in single center cohort, our study is one of rare but valuable studies investigating the necessity for repeat biopsies for Asians with ASAP. In addition, the fact that csPCa was found in about 20% of patients who underwent repeat biopsy might support the evidence that you could not delay repeat biopsy.

45.1% were found with PCa on repeat biopsy in our study and recent literature has reported consistent results for PCa detection rate similar to ours. Oderda et al.^11^ have reported that the rate of PCa in patients with ASAP is 54% at follow-up of 124 months. They concluded ASAP is a strong risk factor for a subsequent PCa, advising a rebiopsy, possibly within 3 months. Another study^12^ has reported that patients with ASAP underwent repeat biopsy within 6 months with 34.5% of PCa detection rate. They showed immediate repeat biopsy remains the correct strategy in absence of predictor factors and non-invasive diagnostic evaluations. Some study^13^ has reported that PCa detection rate is 40.2% in men with ASAP. Prathibha et al.^14^ also found PCa in 42% of ASAP upon repeat biopsy. Once again, our PCa detection rate of 45.1% is very consistent with the recent literature^11–14^ that strengthens our study.

19.6% were found with csPCa on repeat biopsy in our study and consistent findings on csPCa detection rate from ASAP were drawn by several studies. Burks et al.^13^ has reported that GS ≥ 7 PCa detection rate is 22.5%. Prathibha et al.^14^ also found GS ≥ 7 in 17% of high grade prostatic intraepithelial neoplasia (HGPIN) and/or ASAP upon repeat biopsy. Warlick et al.^15^ have reported that 17.3% of men have GS ≥ 7 following a TRUS biopsy for ASAP. Yoshida et al.^16^ reported relatively high csPCa detection rate (13/16 PCa patients, 81.6%) but their study resulted from smaller number of total PCa patients than ours. They indicated that decision making for a repeat biopsy could be affected by clinical characteristics like a small prostate (*p* = 0.0250) and advanced age (*p* = 0.0297) in real-world settings. Once again, our csPCa detection rate of 19.6% is very consistent with the recent literature^13–15^ that strengthens our study.

Although there are not many studies that analyzed the results extensively from ASAP to AP at RP at once, our finding (20% of AP at RP) is comparable to several previous studies. One study^12^ found 35% (7/20) patients were upgraded from non-csPCa to csPCa according to modified Epstein Criteria (clinically organ confined disease, GS sum ≤ 6, up to two positive cores, 50% of core involved with tumor, PSA < 10 ng/mL)^17,18^. Dorin et al.^6^ observed up to 51% of patient presented csPCa according to modified Epstein Criteria, whereas 38% of patients who finally underwent RP had GS ≥ 7 in RP specimen (14% of tumors revealed pT3a). Schiavina et al.^19^ revealed 22.5% of patients harbored unfavorable disease (non-organ confined disease or GS ≥ 4 + 3) at final pathology and the presence of ASAP was independently associated with a higher risk of unfavorable disease (hazard ratio 5.358, 95% confidence interval 1.683–17.0054, *p* = 0.004).

This study has several limitations. First of all, the main limitation is the length of the study cohort with corresponding different management and procedures. Variability in time to repeat biopsy for ASAP also demonstrated the lack of uniformity in practice patterns within single institution. Moreover, as factors such as extended biopsy and MRI fusion biopsy (started in 2015, near the end of the study period) were likely not available for the entire study regarding the long study period, clinicians should be cautious with interpreting our findings. Second, our cohort of 102 patients with repeated biopsies was small. In addition, this study was retrospective in nature. Nevertheless, ASAP is a relatively rare entity. Our inclusion criteria were applied to an initial cohort of more than 3000 men who underwent prostate biopsy. In addition, interobserver variability in the diagnosis of ASAP might not be fully eliminated, although our pathologists reviewed all cases and confirmed the diagnosis of ASAP prior to their inclusion. Although we reported a higher MRI fusion biopsy rate in the repeat biopsy group, there is a possibility of time bias as MRI biopsies were not available at the beginning of the cohort, which clinicians should consider when applying to their practice. Lastly, we could not clearly elucidate predictors of PCa or csPCa from ASAP patients with rebiopsy. This might be due to a small number of cohort and the scarce incidence of ASAP. Further prospective studies need to overcome these limitations and incorporate some recent advances in PCa risk stratification such as PHI, new imaging technologies, and genetic biomarkers.

## Conclusions

In our study, 45.1% of patients with an initial diagnosis of ASAP who had repeat prostate biopsy were subsequently diagnosed with PCa and 19.6% were found to have csPCa. Our csPCa detection rate on repeat biopsy is consistent with data in the contemporary literature (17–22.5%) and adds further evidence that after a diagnosis of ASAP, a repeat biopsy is warranted and that the repeat biopsy should not be postponed. The use of biomarker for improving the specificity of screening and/or mpMRI can also be considered in these patients.

## Supplementary Information

Supplementary Table S1.
