# Supplementary material for: Clinical strategy of repeat biopsy in patients with atypical small acinar proliferation (ASAP)
Source: Sci Rep. 2021 Nov 30;11:23143. doi: 10.1038/s41598-021-02172-8 (PMC8633016; doi:10.1038/s41598-021-02172-8)
Supplement: Supplementary file 1 — Supplementary Table S1. [file 41598_2021_2172_MOESM1_ESM.docx]

Supplementary Table S1. Comparative analysis between repeat biopsy group and non-repeat biopsy group

| Variables | **Repeat biopsy** | | **p value** |
| --- | --- | --- | --- |
|  | **Yes (N=102)** | **No (N=110)** |  |
| Age (Mean ± SD) | 65.0 ±7.3 | 66.4 ± 8.9 | 0.214 |
| BMI (Mean ± SD) | 24.3 ± 2.6 | 24.9 ± 2.8 | 0.138 |
| Diabetes mellitus | 16 (15.7%) | 16 (14.5%) | 0.817 |
| Initial PSA (Mean ± SD) | 7.81 ± 5.30 | 8.56 ± 12.37 | 0.575 |
| PSAD (Mean ± SD) | 0.19 ± 0.14 | 0.19 ± 0.21 | 0.906 |
| Number of Bx core (Mean ± SD) | 12.3 ± 0.7 | 12.5 ± 0.8 | 0.141 |
| Prostate MRI after 1^st^ biopsy | 25 (22.7%) | 25 (24.5%) | 0.760 |
| Suspicious nodules at prostate MRI | 18 (17.6%) | 4 (3.6%) | <0.001 |
| MRI Fusion biopsy | 19 (18.5%) | 5 (4.5%) | 0.001 |

SD: Standard deviation; BMI: Body mass index; PSA: Prostate specific antigen (ng/mL); PSAD: Prostate specific antigen density (ng/mL/cc); Bx: prostate biopsy. MRI: Magnetic resonance imaging
